# Hepatitis B Virus X Protein Drives Multiple Cross-Talk Cascade Loops Involving NF-κB, 5-LOX, OPN and Capn4 to Promote Cell Migration

**DOI:** 10.1371/journal.pone.0031458

**Published:** 2012-02-15

**Authors:** Xuan Zhang, Xiaona You, Qi Wang, Tao Zhang, Yumei Du, Na Lv, Zhao Zhang, Shuai Zhang, Changliang Shan, Lihong Ye, Xiaodong Zhang

**Affiliations:** 1 Department of Cancer Research, Key Laboratory of Molecular Microbiology and Technology of Ministry of Education, Institute for Molecular Biology, College of Life Sciences, Nankai University, Tianjin, People's Republic of China; 2 Department of Biochemistry, College of Life Sciences, State Key Laboratory of Medicinal Chemical Biology, Nankai University, Tianjin, People's Republic of China; Yonsei University, Korea, Republic of

## Abstract

Hepatitis B virus X protein (HBx) plays an important role in the development of hepatocellular carcinoma (HCC). However, the mechanism remains unclear. Recently, we have reported that HBx promotes hepatoma cell migration through the upregulation of calpain small subunit 1 (Capn4). In addition, several reports have revealed that osteopontin (OPN) plays important roles in tumor cell migration. In this study, we investigated the signaling pathways involving the promotion of cell migration mediated by HBx. We report that HBx stimulates several factors in a network manner to promote hepatoma cell migration. We showed that HBx was able to upregulate the expression of osteopontin (OPN) through 5-lipoxygenase (5-LOX) in HepG2-X/H7402-X (stable HBx-transfected cells) cells. Furthermore, we identified that HBx could increase the expression of 5-LOX through nuclear factor-κB (NF-κB). We also found that OPN could upregulate Capn4 through NF-κB. Interestingly, we showed that Capn4 was able to upregulate OPN through NF-κB in a positive feedback manner, suggesting that the OPN and Capn4 proteins involving cell migration affect each other in a network through NF-κB. Importantly, NF-κB plays a crucial role in the regulation of 5-LOX, OPN and Capn4. Thus, we conclude that HBx drives multiple cross-talk cascade loops involving NF-κB, 5-LOX, OPN and Capn4 to promote cell migration. This finding provides new insight into the mechanism involving the promotion of cell migration by HBx.

## Introduction

Hepatitis B virus (HBV) has oncogenic potential in the development of hepatocellular carcinoma (HCC). The HBV genome is a partially double-stranded DNA molecule with four open reading frames (ORFs), in which S ORF encodes hepatitis surface antigen (HBs) and C ORF encodes hepatitis core antigen (HBc). Hepatitis B virus X protein (HBx) is a 17 kDa protein encoded by the X ORF, which plays a crucial role in hepatocarcinogenesis [1]. HBx has multiple biological functions, including interaction with other proteins, mediation of cell proliferation and apoptosis [2], [3]. Recent studies have shown that HBx is associated with cell migration, implicating HBx in HCC metastasis. For example, HBx may promote tumor spreading by facilitating integrin-mediated cell migration and regulating the adhesion-deadhesion balance of the cells in the primary tumor site [4], enhancing CD44-mediated HA-interaction efficiency and modifying the migratory properties of transformed hepatocytes [5] and inducing matrix metalloproteinase (MMP) activation [6], [7], [8]. It has been reported that 5-lipoxygenase (5-LOX) is a key regulator of malignant mesothelial cell proliferation and survival via a VEGF-related circuit [9]. Our laboratory previously found that cyclooxygenase-2 (COX-2) and 5-LOX were highly expressed in breast cancer LM-MCF-7 cells and MDA-MB-231 cells, which were related to breast cancer metastasis [10]. Moreover, our have found that HBx could upregulate the levels of cyclooxygenase-2 (COX-2) and 5-lipoxygenase in liver cells [11]. Accordingly, nuclear factor-κB (NF-κB) plays an instrumental role in carcinogenesis and in the regulation of immune and inflammatory responses [12]. NF-κB induces the expression of various target genes related to proliferation, apoptosis, angiogenesis and metastasis. HBx protein activates the transcription factor NF-κB by acting on two distinct cytoplasmic NF-κB inhibitor pathways [13]. Furthermore, HBx can induce the expression of various target genes through activation of NF-κB, such as cyclin D1 through the NF-κB2(p52)/BCL-3 complex in the nucleus [14]. HBx induces expression of the CXC chemokine IP-10 and MIG and increases migration of leukocytes through the activation of NF-κB [15], [16].

Previous studies demonstrated that tumor cell invasion and metastasis after liver transplantation for HCC was highly correlated with overexpression of calpain small subunit 1 (Capn4) [17], which belongs to the calpain system [18]. Recently, we have reported that HBx could promote hepatoma cell migration through the upregulation of Capn4 [19]. Several reports have revealed that osteopontin (OPN) plays important roles in tumor cell adhesion, migration, invasion and angiogenesis [20], [21], [22], [23]. An elevated level of plasma OPN is significantly related to cancer invasiveness and has a significant impact on tumor development and patient survival rate [24]. OPN is overexpressed in multiple tumor tissues and is associated with invasion, progression or metastasis in numerous human cancers, such as liver [25], breast and colon [26] cancer. OPN promotes tumor cell migration via the regulation of multiple signaling pathways and activation of metastasis-related gene expression. Some downstream effectors of OPN, including PI3K/Akt, EGFR, HGFR, MMPs, and NF-κB, mediate critical metastatic processes [27], [28], [29], [30], [31]. Therefore, we hypothesize that 5-LOX, NF-κB/p65 and OPN may be involved in cell migration mediated by HBx and Capn4.

In the present study, we investigated the signal pathways involving hepatoma cell migration promoted by HBx. Our finding shows that HBx drives multiple cross-talk cascade loops to promote hepatoma cell migration, providing new insight into the mechanism of development of HBx-mediated HCC.

## Results

### HBx upregulates the expression of OPN in hepatoma cells

To investigate whether HBx upregulates the expression of OPN, we examined the effect of HBx on the promoter activity of OPN. Our data showed that HBx could significantly enhance the promoter activity of OPN by luciferase reporter gene assay in a dose dependent manner (Figure S1A, see Text S1). Meanwhile, RNA interference (RNAi) targeting HBx mRNA mediated by pSilencer3.0-X (pSi-HBx) could abolish the increase of OPN promoter activity in a dose-dependent manner (Figure S1A, see Text S1). In addition, western blot analysis showed that HBx upregulated the expression of OPN in HepG2-X (or H7402-X) cells, respectively (Figure S1B, see Text S1). The knock down of HBx could attenuate the upregulation of OPN in the cells by pSi-HBx in a dose-dependent manner (Figure S1B, see Text S1). Thus, we conclude that HBx is able to upregulate the expression of OPN.

### 5-LOX is responsible for the upregulation of OPN mediated by HBx

**Figure 1:**
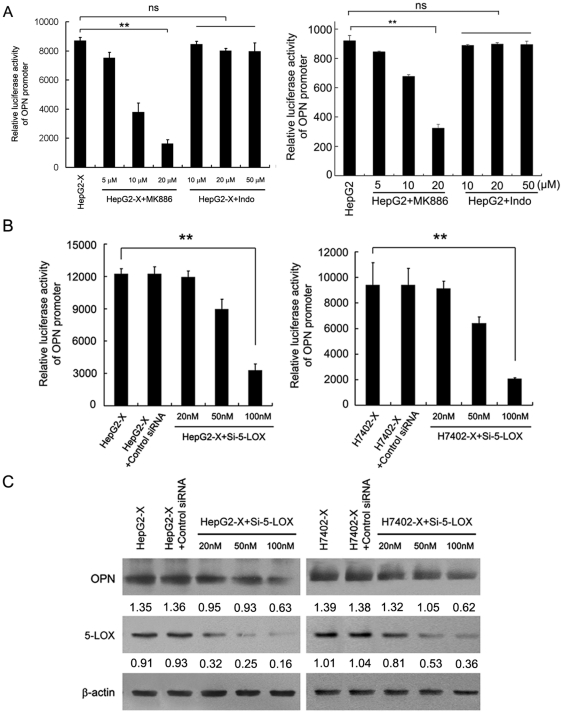
5-LOX is responsible for the upregulation of OPN mediated by HBx. (A) The promoter activities of OPN were detected by luciferase reporter gene assay in HepG2-X and HepG2 cells treated with MK886 or Indo, respectively (**P<0.01, ns, not significant, Student's *t* test). (B) The promoter activity of OPN was detected by luciferase reporter gene assay in HepG2-X (or H7402-X) cells treated with the indicated doses of siRNA targeting 5-LOX mRNA (Si-5-LOX) (**P<0.01, Student's *t* test). (C) The protein levels of OPN, 5-LOX and HBx were examined in the cells by western blot analysis.

5-LOX, a catalyzing enzyme of arachidonic acid, was overexpressed during multistage tumor progression in many neoplastic disorders including lung, breast, pancreatic cancers and HCC HBx-integrated host hepatocytes [32], [33]. Our previous study found that HBx could upregulate 5-LOX and COX-2, which were involved in proliferation and migration [10], [11]. Therefore, we hypothesize that 5-LOX or COX-2 may be involved in the HBx-mediated OPN upregulation. To answer this question, we treated HepG2-X cells with MK886 (an inhibitor of 5-LOX) and Indo (an inhibitor of COX-2) for 6 h. Luciferase reporter gene assays demonstrated that MK886 could abolish the enhanced promoter activity of OPN in HepG2-X cells in a dose-dependent manner (Figure 1A). However, the treatment with Indo failed to affect the promoter activity of OPN (Figure 1A). In addition, we found that 5-LOX was also able to upregulate the promote activity of OPN in HepG2 cells, but the luciferase activity was lower than that in HepG2-X cells (Figure 1A), suggesting that HBx upregulates OPN through 5-LOX. Furthermore, we found that siRNA against 5-LOX (Si-5-LOX) could abolish the increased promoter activity and upregulated expression of OPN protein in HepG2-X (or H7402-X) cells in a dose-dependent manner by luciferase reporter gene assay (Figure 1B) and western blot analysis (Figure 1C). Thus, we conclude that HBx is able to upregulate the expression of OPN through 5-LOX.

Next, we examined the effect of HBx on the regulation of 5-LOX. We confirmed that the 5-LOX expression levels were markedly upregulated in HepG2-X (H7402-X) cells relative to the HepG2 (H7402) cells by real-time PCR and western blot (Figure S2A, S2B, see Text S1), which is consistent with our previous study [11]. HBx RNAi could abolish the upregulation of 5-LOX (Figure S2A, S2B, see Text S1). Furthermore, the data showed that the amount of LTB4, a metabolite of 5-LOX, was higher in the conditioned media of HepG2-X (or H7402-X) cells than that in HepG2 (or H7402) cells, which could be abolished by RNAi targeting HBx mRNA (Figure S2C, D, see Text S1).

### NF-κB is responsible for the upregulation of 5-LOX mediated by HBx

**Figure 2:**
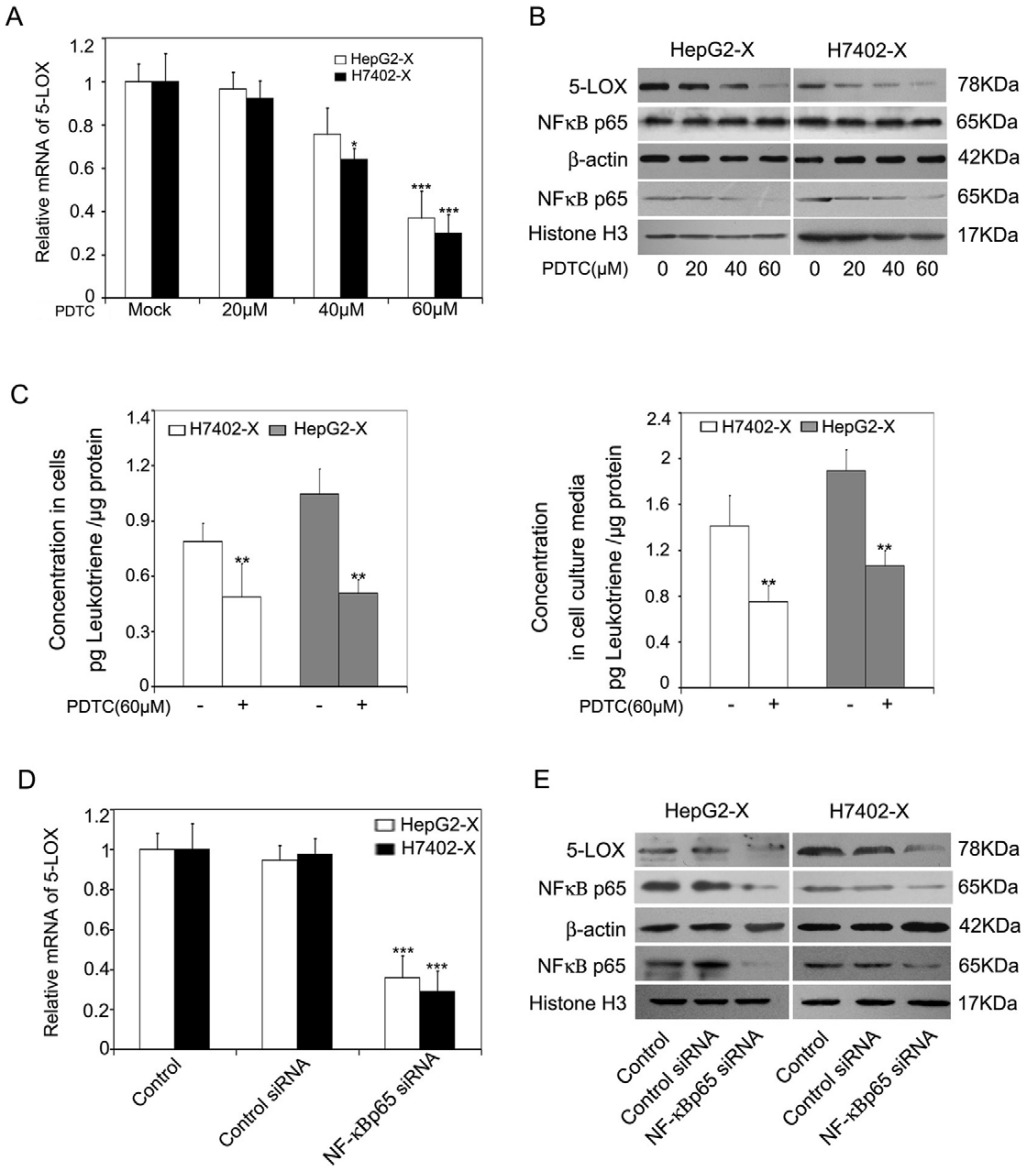
NF-κB is responsible for the upregulation of 5-LOX. (A, B and C) HepG2-X (or H7402-X) cells were treated with PDTC (0, 20, 40 and 60 µM) for 6 h. (A) The mRNA levels of 5-LOX were examined by real-time PCR (*P<0.05, ***P<0.001, Student's *t* test). (B) The protein levels of 5-LOX and NF-κB/p65 were examined by western blotting. (C) The amount of LTB4 was detected by ELISA in conditioned media or in cell lysates (**P<0.01, Student's *t* test). (D and E) HepG2-X (or H7402-X) cells were transfected with siRNA targeting NF-κB/p65 mRNA or control siRNAs for 48 h. (D) The mRNA levels of 5-LOX were measured by real-time PCR (***P<0.001, Student's *t* test). (E) The protein levels of 5-LOX and NF-κB/p65 were examined by western blot analysis.

Our previous study reported that HBx activates NF-kB/p65 in HepG2-X and H7402-X cell lines [19]. Moreover, we found that 60 µM PDTC (an inhibitor of NF-κB) could significantly downregulate the expression of 5-LOX mRNA and protein in HepG2-X (or H7402-X) cells (Figure 2A and B). In addition, 60 µM PDTC could decrease the amount of released LTB4 in the conditioned media of HepG2-X (or H7402-X) cells and cell lysates (Figure 2C). Meanwhile, 100 nM siRNA against NF-κB p65 was able to attenuate the levels of mRNA and protein of 5-LOX in HepG2-X (or H7402-X) cells (Figure 2D and E), which was consistent with the above data. In the control, immunoblot analysis showed that 60 µM PDTC (or 100 nM NF-κB p65 siRNA) could successfully downregulate the expression of NF-kB/p65 in nuclear extracts from HepG2-X (or H7402-X) cells (Figure 2B and E). Thus, our data suggest that NF-κB is responsible for the HBx-mediated upregulation of 5-LOX.

### OPN is responsible for the HBx-mediated upregulation of Capn4

**Figure 3:**
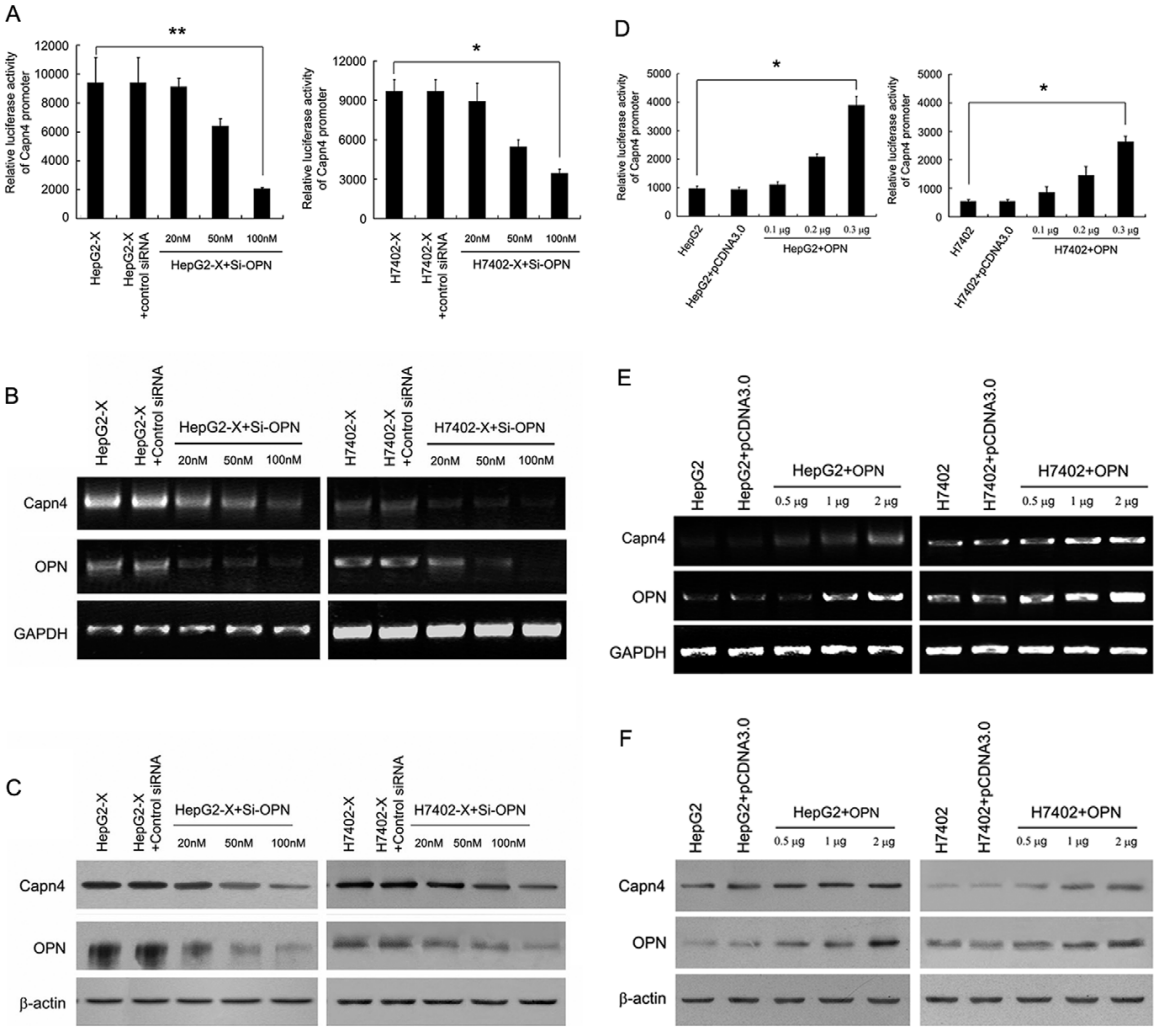
OPN is responsible for the HBx-mediated upregulation of Capn4. (A, B and C) HepG2-X (or H7402-X) cells were transfected with the indicated doses of siRNA targeting OPN mRNA (Si-OPN) and control siRNA for 48 h. (A) The promoter activity of Capn4 was detected by luciferase reporter gene assay (**P<0.01, *P<0.05 Student's *t* test). (B) The mRNA levels of Capn4 were detected by RT-PCR. (C) The protein levels of Capn4 were examined by western blot analysis. (D, E and F) HepG2 (or H7402) cells were transiently transfected with the indicated doses of pcDNA3.0-OPN for 48 h. (D) The promoter activity of Capn4 was measured by luciferase reporter gene assay (*P<0.05 Student's *t* test). (E, F) The mRNA and protein levels of Capn4 were examined by RT-PCR and western blot analysis, respectively.

Recently, we have reported that HBx upregulates the expression of Capn4 through NF-κB to promote cell migration [19]. Previous studies demonstrated that OPN was significantly associated with tumor metastasis by activating other metastasis-related genes. Therefore, we hypothesize that OPN may be correlated with the HBx-mediated upregulation of Capn4. To address this question, we examined the effect of OPN knockdown (Si-OPN) on the promoter activity of Capn4 in HepG2-X (or H7402-X) cells by luciferase reporter gene assay. The results showed that Si-OPN could abolish the enhanced promoter activity of Capn4 in a dose-dependent manner (Figure 3A). Furthermore, we detected an effect of Si-OPN on the expression of Capn4 at the mRNA and protein level in HepG2-X (or H7402-X) cells by RT-PCR and western blot analysis. The data revealed that Si-OPN could significantly attenuate the HBx-mediated upregulation of Capn4 at the mRNA and protein levels in a dose-dependent manner (Figure 3B and C). This suggests that OPN is responsible for the HBx-mediated upregulation of Capn4. To further confirm the role of OPN in the upregulation of Capn4, we examined the effect of OPN on the expression of Capn4 by transient transfection of pcDNA3.0-OPN in HepG2 (or H7402) cells. Luciferase reporter gene assays showed that the overexpression of OPN led to a strong enhancement of promoter activity of Capn4 in a dose-dependent manner in HepG2 (or H7402) cells (Figure 3D). Furthermore, Capn4 mRNA and protein levels were increased in the cells by the overexpression of OPN in a dose-dependent manner (Figure 3E and F). Our data suggest that OPN is able to stimulate the expression of Capn4.

### NF-κB is responsible for the OPN-mediated upregulation of Capn4

**Figure 4:**
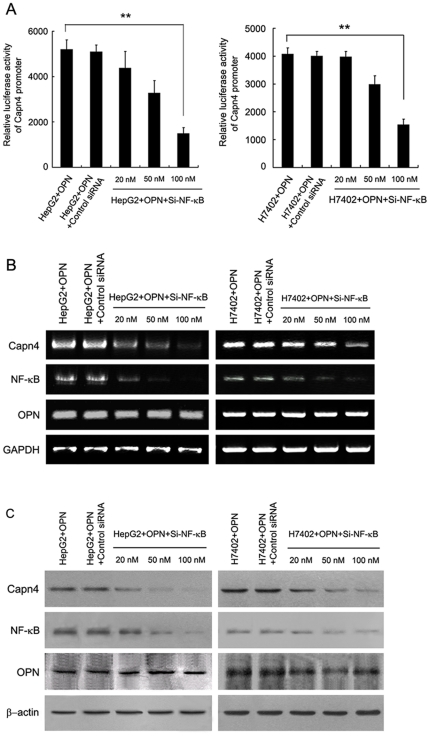
NF-κB is responsible for the OPN-mediated upregulation of Capn4. (A, B and C) OPN-overexpressed HepG2 (or H7402) cells were treated with the indicated doses of siRNA targeting NF-κB mRNA (Si-NF-κB) for 48 h. (A) The promoter activity of Capn4 was examined by reporter gene assay (**P<0.01 Student's *t* test). (B) The mRNA levels of Capn4, OPN and NF-κB/p65 were detected by RT-PCR. (C) The protein levels of Capn4, OPN and NF-κB/p65 were measured by western blot analysis.

It is known that OPN induces NF-κB activation, resulting in the upregulation of its downstream effectors via attachment to β-3 integrin receptors and the induction of cell spreading and activation [28], [30], [34]. Therefore, we speculated that NF-κB may be responsible for the OPN-induced upregulation of Capn4. Luciferase reporter gene assays revealed that knockdown of NF-κB (Si-NF-κB) resulted in the abolishment of enhanced OPN-mediated promoter activity of Capn4 in a dose-dependent manner (Figure 4A). Additionally, the upregulation of Capn4 mediated by the overexpression of OPN could be attenuated by Si-NF-κB at the mRNA and protein levels in a dose-dependent manner (Figure 4B and C). Thus, we conclude that NF-κB is responsible for the OPN-mediated upregulation of Capn4.

### OPN is upregulated by Capn4 in a positive feedback manner

**Figure 5:**
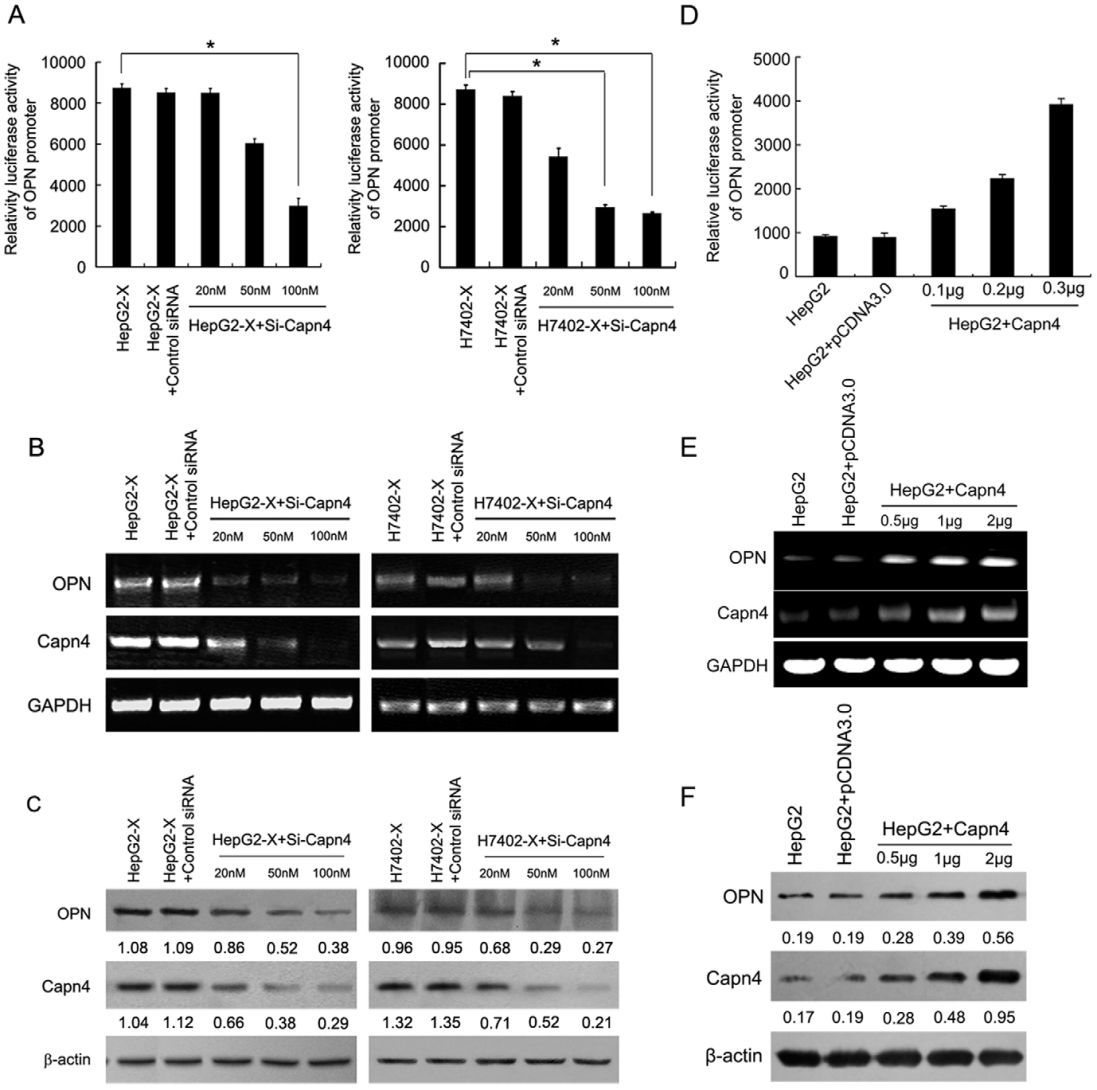
Capn4 can regulate OPN in a positive feedback manner. (A, B and C) HepG2-X (or H7402-X) cells were transfected for 48 h with the indicated doses of siRNA targeting Capn4 mRNA (Si-Capn4). (A) The promoter activity of OPN was measured by luciferase reporter gene assay (*P<0.05, Student's *t* test). (B, C) The mRNA and protein levels of OPN were detected by RT-PCR and western blot analysis, respectively. (D, E and F) HepG2 cells were transiently transfected with the indicated doses of pcDNA3.0-Capn4 for 48 h. (D) The promoter activity of OPN was measured by luciferase reporter gene assay (*P<0.05 Student's *t* test). (E, F) The mRNA and protein levels of OPN were examined by RT-PCR and western blot analysis, respectively.

Previously, we found that the tumor cell maintained proliferation and migration in a positive feedback manner [10], [11], [35]. Therefore, we hypothesize that Capn4 may be involved in the activation of OPN in a positive feedback manner as well. To test the hypothesis, we determined the effect of Capn4 on the regulation of OPN in HepG2-X (or H7402-X) cells by siRNA targeting Capn4 mRNA (Si-Capn4). Interestingly, we found that the enhanced promoter activity of OPN could be abolished by Si-Capn4 in a dose-dependent manner (Figure 5A). Additionally, the expression of OPN at the levels of mRNA and protein could be attenuated by Si-Capn4 in a dose-dependent manner (Figure 5B and C). The data suggest that OPN is regulated by Capn4 in a positive feedback manner in HepG2-X (or H7402-X) cells. To further confirm that Capn4 is able to upregulate OPN, we examined the effect of Capn4 on the expression of OPN by transient transfection of pcDNA3.0-Capn4 in HepG2 cells. Luciferase reporter gene assays showed that overexpression of Capn4 led to a strong enhancement of promoter activity of OPN in a dose-dependent manner in HepG2 cells (Figure 5D). Furthermore, RT-PCR and western blot confirmed that the expression levels of OPN were increased by the overexpression of Capn4 in a dose-dependent manner (Figure 5E and F). Our data suggest that Capn4 is able to stimulate the expression of OPN.

### NF-κB is responsible for the Capn4-mediated upregulation of OPN

**Figure 6:**
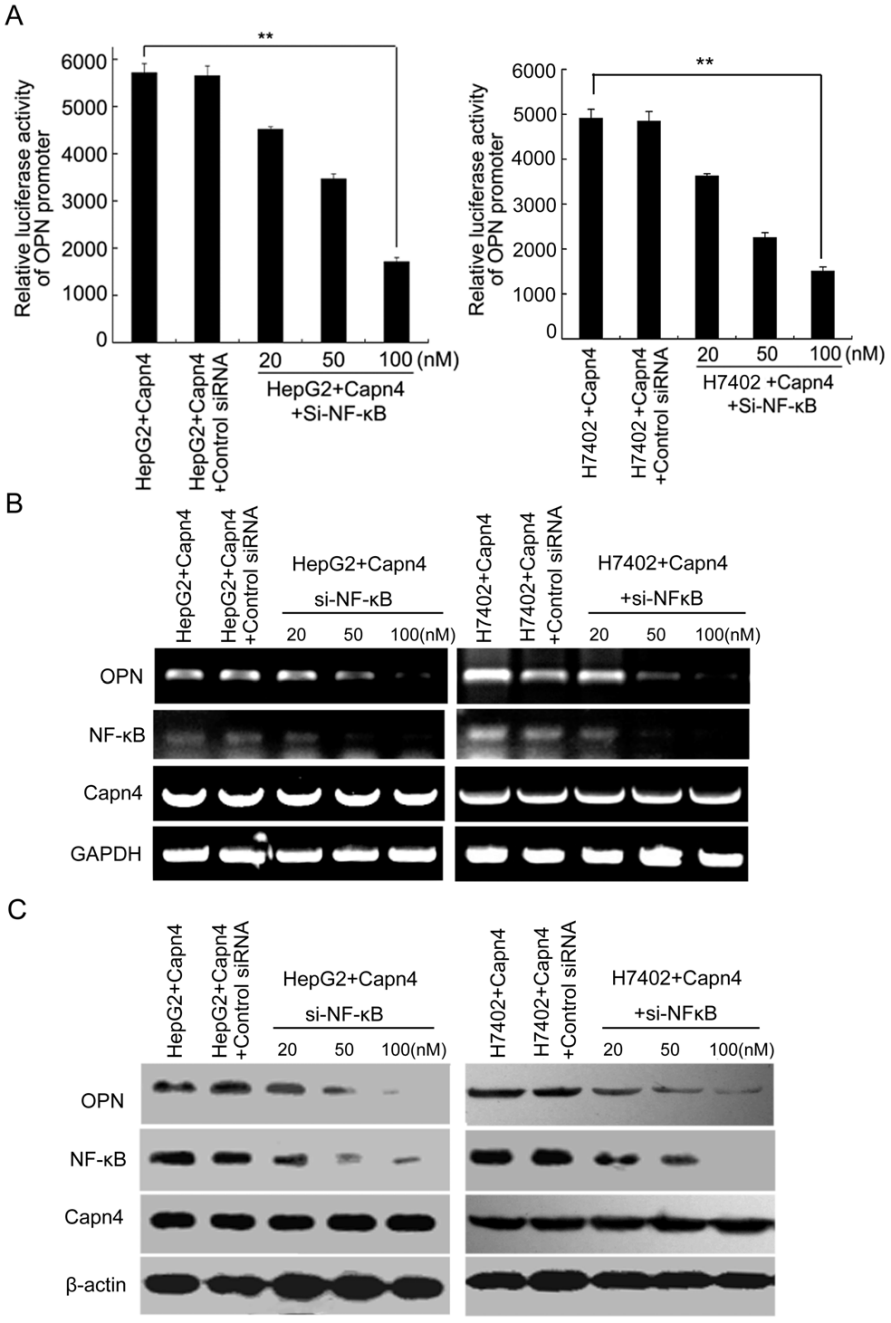
NF-κB is responsible for the Capn4-mediated upregulation of OPN. (A, B and C) Capn4-overexpressed HepG2 (or H7402) cells were treated with the indicated doses of siRNA targeting NF-κB mRNA (Si-NF-κB) for 48 h. (A) The promoter activity of OPN was examined by luciferase reporter gene assay (**P<0.01 Student's *t* test). (B) The mRNA levels of OPN, Capn4 and NF-κB/p65 were detected by RT-PCR. (C) The protein levels of OPN, Capn4 and NF-κB/p65 were measured by western blot analysis.

It has been reported that Capn4 activates NF-κB in HeLa cells, which is involved in the survival pathway [36]. Therefore, we speculated that NF-κB may be involved in the Capn4-induced upregulation of OPN. Luciferase reporter gene assays revealed that the knockdown of NF-κB (Si-NF-κB) resulted in the abolishment of enhanced promoter activity of OPN mediated by Capn4 in a dose-dependent manner (Figure 6A). Additionally, the upregulation of Capn4 mediated by OPN could be attenuated by Si-NF-κB at the levels of mRNA and protein in a dose-dependent manner (Figure 6B and C). Thus, we conclude that NF-κB is responsible for the Capn4-mediated upregulation of OPN.

### OPN, Capn4 and NF-κB are involved in HBx-mediated hepatoma cell migration

Furthermore, we examined the effect of OPN and Capn4 on the migration of hepatoma cells mediated by HBx. Wound healing assays showed that HepG2-X cells exhibited a much greater ability to repair the wound compared to control cells. However, the migration ability of HepG2-X cells could be suppressed by the treatment with siRNAs targeting HBx, OPN, Capn4 and NF-κB (Figure S5A, B and Figure S6A, B, see Text S1), respectively. The transient transfection of pCMV-X significantly promoted the migration of HepG2 cells, but the transient transfection of pCMV-HBc or pCMV-HBs failed to enhance the migration. In addition, the transient transfection of pcDNA-OPN and pcDNA-Capn4 also promoted the migration of HepG2 cells (Figure S5C, S5D, see Text S1), suggesting that OPN and Capn4 are involved in the promotion of HBx-mediated hepatoma cell migration.

## Discussion

Recent studies have shown that HBx is associated with cell migration. Moreover, our previous studies demonstrated that HBx is able to promote cell migration through the regulation Capn4 or mir-29a [19], [37]. However, the mechanisms of promotion of cell migration mediated by HBx are not fully understood. In the present study, we investigated the signaling pathways of hepatoma cell migration mediated by HBx. Studies demonstrate that OPN is closely related to tumor metastasis and is often overexpressed in multiple tumor tissues and correlated with metastatic tissues [22], [23]. Thus, we first tested whether HBx was able to upregulate OPN. The data demonstrated that HBx significantly upregulated OPN in HepG2-X (or H7402-X) cells. Our laboratory previously found that 5-LOX and COX-2 were involved in cell proliferation and migration [10], [11]. Accordingly, 5-LOX is often overexpressed in multiple tumor progression [33]. Therefore, we hypothesized that 5-LOX or COX-2 may be involved in the HBx-mediated upregulation of OPN. Interestingly, we showed that HBx could induce the upregulation of OPN in HepG2-X (or H7402-X) cells through 5-LOX, rather than COX-2 (Figure 1). We then examined the mechanism of HBx-mediated upregulation of 5-LOX. It has been reported that the constitutive activation of NF-κB is prevalent in liver tumor tissues [38]. NF-κB is one of important transcription factors regulated by HBx, which induces the expression of various target genes with various functions, such as, proliferation and migration [13], [14], [15], [16]. Therefore, we speculated that NF-κB may be involved in the regulation of 5-LOX expression mediated by HBx. Our results demonstrated that the treatment with PDTC (a specific NF-κB inhibitor) and siRNAs against NF-κB abolished the HBx-mediated upregulation of 5-LOX (Figure 2), suggesting that NF-κB is responsible for the upregulation of 5-LOX.

Our laboratory reported that HBx can upregulate Capn4 through NF-κB in the promotion of hepatoma cell migration [19]. Capn4 is a regulated subunit of calpains, and plays important roles in the regulation of intracellular activities, including cell spreading and migration [39], proliferation, apoptosis and differentiation [40]. Accordingly, OPN can promote tumor cell migration via the regulation of multiple signaling pathways and activation of metastasis-related gene expression. Thus, we supposed that OPN may be involved in the activation of Capn4 mediated by HBx. Our data confirmed that OPN was responsible for the upregulation of Capn4 in HepG2-X (or H7402-X) cells (Figure 3). Several studies have reported that OPN can promote down-stream effectors, such as metastasis-related genes, through the activation of the NF-κB pathway [28], [30]. We next examined whether NF-κB was involved in the OPN-mediated upregulation of Capn4. The results showed that NF-κB was responsible for the event (Figure 4). HBx frequently regulates signal transduction in a feedback manner [35]. Then, we examined that whether Capn4 is involved in the activation of OPN. Our data showed that Capn4 was able to upregulate OPN in a positive feedback manner (Figure 5). NF-κB plays an important role in regulation of 5-LOX and OPN mediated by HBx as above. Thus, we try to demonstrate whether Capn4 regulates OPN through NF-κB as well. Our finding showed that NF-κB was also involved in the Capn4-mediated upregulation of OPN indeed (Figure 6), suggesting that NF-κB is an important factor in regulation of proteins involving cell migration. To show that the above observations are relevant to HBx expression during an HBV infection, we further examined the effect of HBx on regulation of NF-κB, 5-LOX, OPN and Capn4 in HepG2.2.15 cells using HBx RNAi. We found that HBx was able to upregulate the expression of the proteins in the cells (Figure S3). Meanwhile, we showed that the overexpression of hepatitis B virus surface antigen (HBsAg) gene or hepatitis B virus core antigen (HBcAg) gene failed to affect the promoter activities of OPN and Capn4 (Figure S4), supporting that HBx is responsible for the upregulation of NF-κB, 5-LOX, OPN and Capn4. In function, we revealed that those factors such as OPN, Capn4 and NF-κB were involved in hepatoma cell migration promoted by HBx (Figure S5, S6). Interestingly, our finding showed that a multiple factors such as NF-κB, 5-LOX, OPN and Capn4 were involved in the regulation of cell migration meditated by HBx with a cascade signaling transduction, in which NF-κB was responsible for regulation of all other three factors. Growing evidence reported that HBx, OPN and Capn4 were able to activate transcription factor NF-κB [13], [28], [30], [36]. Importantly, OPN and Capn4 affected each other through NF-κB in a positive feedback loop. The data suggested that those factors regulate cell migration in a network manner. Thus, HBx drives a multiple signaling, such as, NF-κB, 5-LOX, OPN and Capn4, in positive feedback loop manner to promote hepatoma cell migration.

Taken together, we conclude that HBx can enhance hepatoma cell migration through the activation of NF-κB, 5-LOX, OPN and Capn4 cascade loops with multiple cross-talk events, which contribute to the sustained promotion of cell migration mediated by HBx. Moreover, NF-κB plays an important role in the cascade loops of signaling pathways. Our finding provides new insight into the mechanism of hepatoma cell migration promoted by HBx.

## Materials and Methods

### Plasmids, reagents and siRNAs

The plasmids pCMV-X, pcDNA3.0, pSilencer3.0-X, pGL3-Basic, pGL3-control, pGL3-Capn4 and renilla luciferase reporter vector pRL-TK were previously described [19], [41]. The pGL3-OPN plasmid was the firefly luciferase reporter plasmid containing the full-length OPN promoter sequence [42]. The pcDNA3.0-OPN and pcDNA3.0-Capn4 plasmid were the eukaryotic expression vector containing the full-length OPN or Capn4 mRNA sequence. MK886 (an inhibitor of 5-LOX), Pyrrolidine dithiocarbamate (PDTC, an inhibitor of NF-κB) and indomethacin (Indo, an inhibitor of COX-2) were purchased from Sigma-Aldrich (USA). The enzyme immunoassay kit used for measurement of leukotriene B4 (LTB4) was purchased from Adlitteram Diagnostic Laboratories (USA). The siRNAs targeting the human mRNA of OPN (targeting sequence: 5′-GCCACAAGCAGTCCAGATT-3′; D28759) [43], 5-LOX (targeting sequence: 5′-GCGCAAGTACTGGCTGAATGA-3′; NM_000698), NF-κB/p65 (targeting sequence: 5′-ACAAGGTGCAGAAAGAGGACA-3′; NM_021975) [44], Capn4 (targeting sequence: 5′-GCTTTTGTTCTCTCAGTAC-3′; NM_001749) [13] and the negative control siRNA were designed and synthesized by RiboBio (Guangzhou, China).

### Cell culture

Human hepatoma HepG2 and H7402 cells, HepG2-P/H7402-P cells (stably transfected with the empty pCMV-Tag2B vector plasmid) and HepG2-X/H7402-X cells (stably transfected with the pCMV-X plasmid) were maintained in Dulbecco's modified Eagle's (DMEM) medium (Gibco, USA) supplemented with heat inactivated 10% fetal calf serum (FCS), 100 U/ml penicillin, and 100 mg/ml streptomycin in a humidified atmosphere of 5% CO_2_ and 95% air at 37°C [35].

### Construction of the human OPN and Capn4 eukaryotic expression plasmid

The full-length human OPN cDNA (942 bp, Gen ID: 6696) and human Capn4 cDNA (801 bp, Gen ID: 826) were cloned into the *EcoRI* and *XhoI* sites of the eukaryotic expression vector pcDNA3.0, using human cDNA as a template, termed pcDNA3.0-OPN or pcDNA3.0-Capn4, respectively. The primers used are listed in table S1.

### RNA interference (RNAi)

HepG2-X (or H7402-X) cells were transfected with the pSilencer-X vector that produces siRNA that targets the HBx mRNA or with control siRNA [41]. Duplex siRNAs targeting the human mRNA of OPN, 5-LOX and Capn4 were introduced into HepG2-X (or H7402-X) cells according to the manufacturer's instructions. Duplex siRNA targeting the human NF-κB/p65 mRNA were transfected into HepG2 (or H7402) cells with overexpression of OPN according to the manufacturer's instructions. Each experiment included controls, which contained the transfection reagent and control siRNA. The transfected cells were subjected to luciferase reporter gene assays, RT-PCR and western blot analysis 48 h after the transfection.

### Treatment of tumor cells

HepG2-X cells were cultured in serum-free medium for 12 h. Briefly, the engineered cells were treated with MK886 (5, 10 or 20 µM), Indo (10, 20 or 50 µM) and PDTC (20, 40 or 60 µM) for 6 h. The treated cells were used to perform luciferase reporter gene assays. The examination of cytotoxicity mediated by MK886 and Indo has been performed [35].

### Transfection

Transfection was performed in cells using Lipofectamine 2000 (Invitrogen, USA). In brief, the cells were plated in 6-well or 96-well plates at 50% confluence. For each well, siRNA was added into 250 µl Opti-MEM medium (Gibco), and 5 µl Lipofectamine 2000 was added into 250 µl Opti-MEM medium and mixed well. The mixture was added and incubated for 6 h before the medium was replaced.

### Luciferase reporter gene assays

Cells (2×10^5^) were plated in a 24-well culture plate and transfected with 300 ng reporter plasmid with 50 ng of pRL-TK encoding renilla luciferase. The treated cells were harvested after 48 h and lysed in 1× passive lysis buffer. The luciferase activity was determined using Dual-Luciferase Reporter® Assay System (Promega, USA) on a Luminometer (TD-20/20, Turner Designs) according to the manufacturer's instructions. Promoter activity was analyzed by detecting firefly luciferase activity and normalizing to renilla luciferase activity in each well, as previously described [41]. Each assay was performed in triplicate.

### RNA extraction, RT-PCR and real-time PCR

Total RNA extraction and reverse transcription were carried out as described previously [45]. Synthesized cDNA was used as a template for PCR (primers are listed in table S1). The RT-PCR products were verified by electrophoresis on a 1% ethidium bromide-stained agarose gel. Quantitative real-time PCR was performed using the SYBR® Premix Ex TaqTM II PCR kit (Takara, Japan) by following the manufacturer's instructions. The relative amounts of mRNAs were calculated using the ΔΔCt method [46] with GAPDH as the endogenous reference gene amplified from the samples. All experiments were performed in triplicate.

### Western blot analysis

After indicated treatments, the cells were washed three times with ice-cold phosphate-buffered saline (PBS). The cytosolic extracts were extracted with lysis buffer (62.5 mM Tris–HCl, pH 6.8, 2% SDS, 5% 2-mercaptoethanol, 10% glycerol, and protease inhibitor cocktail). Nuclear extracts were collected using NEPER Nuclear and Cytoplasmic Extraction kit (Pierce Biotechnology, USA). The western blot protocol was described previously [35]. The primary antibodies used were against β-actin (1∶1000 dilution, Sigma-Aldrich, USA), HBx (1∶1000 dilution, Abcam, UK),Capn4 (1∶1000 dilution, Thermo Fisher Scientific, USA), OPN (1∶800 dilution, provided by Dr. Chao Bian from Shanghai institutes for biological sciences, Chinese Academy of Sciences), 5-LOX (Santa Cruz Biotechnology, USA),histone-3 (Cell Signaling Technology, USA) and NF-κB/p65 (1∶400 dilution, Santa Cruz Biotechnology, USA). All experiments were performed in triplicate.

### Enzyme-linked immunosorbent assay (ELISA)

The amount of LTB4 (a metabolite of 5-LOX) was examined by ELISA according to the manufacturer's instructions. The concentration of LTB4 was normalized to the total protein. The protein concentrations in these extracts were determined by a standard protein assay method (Bio-Rad Laboratories, Inc., USA). All experiments were performed in triplicate.

### Statistical analysis

All values are presented as means ± SEM. Each value is the mean of at least three separate experiments in each group. Data were analyzed by comparing two groups using Student's *t* test. *P*<0.05 was considered significant.

## Supporting Information

Figure S1 **HBx upregulates the expression of OPN.** (A) The promoter activity of OPN was examined by luciferase reporter gene assay in HepG2-X (or H7402-X) cells, which was abolished by RNAi targeting HBx mRNA, using the indicated doses of pSilencer3.0-X (pSi-HBx) plasmid (**P<0.01 Student's *t* test). (B) The protein expression of OPN was detected in HepG2-X (or H7402-X) cells by western blot analysis, which was attenuated by pSi-HBx plasmid in a dose-dependent manner.
(TIF)

Figure S2 **HBx upregulates the expression of 5-LOX.** (A) The expression of 5-LOX was examined by real-time PCR in HepG2-X (or H7402-X) cells, which was abolished by using pSilencer3.0-X plasmid (***P<0.001, Student's *t* test). (B) The expression level of 5-LOX and HBx were detected by western blot analysis. (C,D) The level of LTB4, a metabolite of 5-LOX, was determined by ELISA in conditioned media or in cell lysates from HepG2-X (or H7402-X) cells (*P<0.05, **P<0.01, ***P<0.00l, Student's *t* test).
(TIF)

Figure S3 **HBx upregulates NF-κB, 5-LOX, OPN and Capn4 in HepG2.2.15 cells.** The expression levels of NF-κB, 5-LOX, OPN, Capn4 and HBx were detected by western blot analysis.
(TIF)

Figure S4 **HBx increases the promoter activity of OPN and Capn4, but not by HBc and HBs.** (A) The promoter activity of OPN was examined by luciferase reporter gene assay in HepG2 cells (**P<0.01 Student's *t* test). (B) The promoter activity of Capn4 was examined by luciferase reporter gene assay in HepG2 cells (**P<0.01 Student's t test).
(TIF)

Figure S5 **HBx promotes hepatoma cell migration through OPN and Capn4, but not by HBc and HBs.** (A) The migration ability of hepatoma cells was examined by wound healing assay when the cells were treated by pSi-HBx, Si-OPN or Si-Capn4. Black arrows indicate the wound edge closure of monolayer cells. (B) The average migration distances of the wound edge were measured in three independent experiments (*P<0.05, **P<0.01, Student's *t* test). (C) The migration ability of hepatoma cells was examined by wound healing assay when HBx, HBs, HBc, OPN and Capn4 were overexpressed in the cells. Black arrows indicate the wound edge closure of monolayer cells. (D) The average migration distances of the wound edge were measured in three independent experiments (*P<0.05, **P<0.01, Student's *t* test).
(TIF)

Figure S6 **HBx promotes cell migration through NF-κB.** (A) The migration ability of hepatoma cells was examined by wound healing assay when the cells were treated with Si-NF-κB. (B) The average migration distances of the wound edge were measured in three independent experiments (***P<0.001, Student's *t* test).
(TIF)

Table S1 **List of primers used for PCR analysis.**
(DOC)

Text S1 **Supporting information.**
(DOC)
