# Supplementary figures and images for: Hepatitis B Virus X Protein Drives Multiple Cross-Talk Cascade Loops Involving NF-κB, 5-LOX, OPN and Capn4 to Promote Cell Migration
Source: PLoS One. 2012 Feb 15;7(2):e31458. doi: 10.1371/journal.pone.0031458 (PMC3280298; doi:10.1371/journal.pone.0031458)

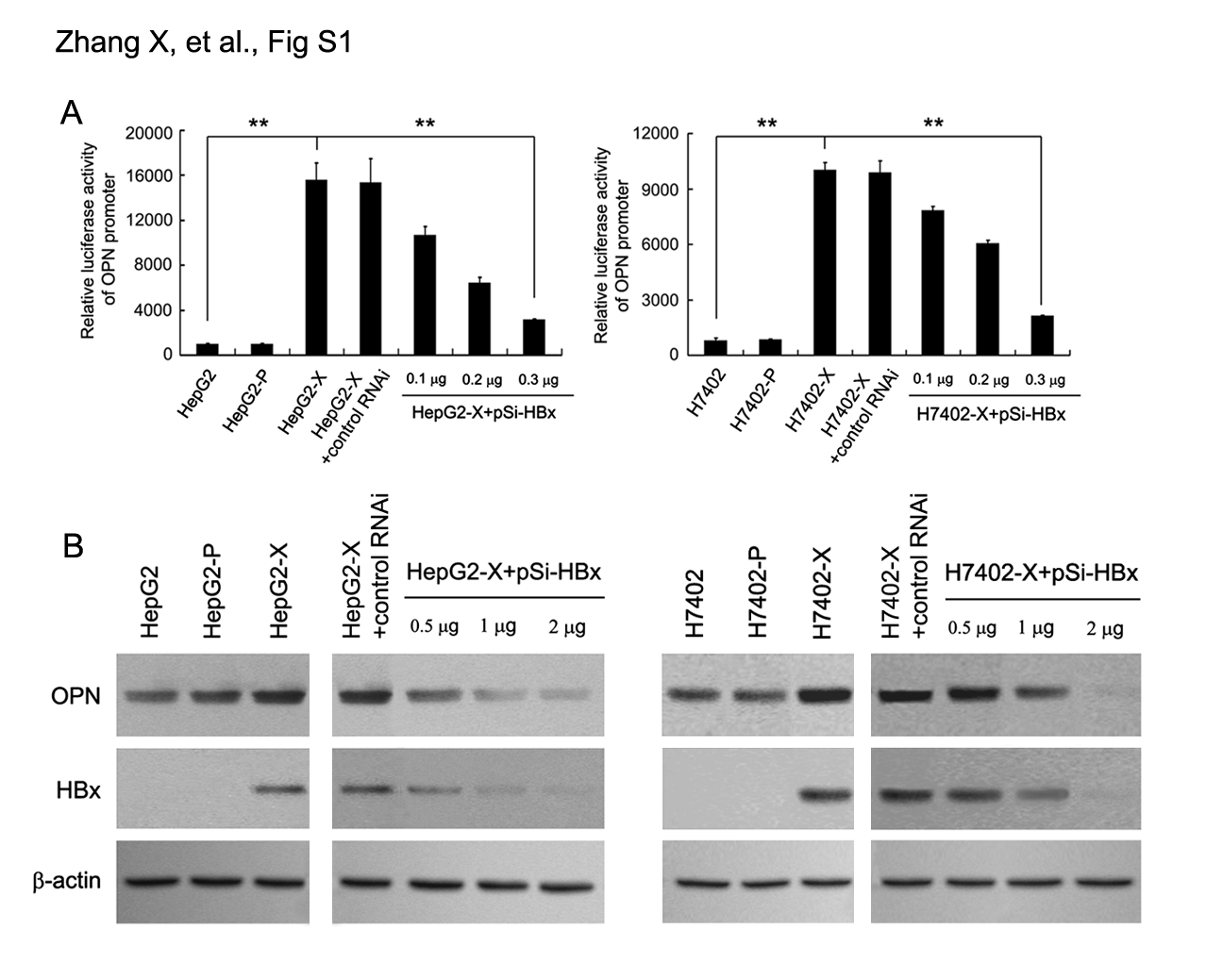

Supplement: Figure S1 — HBx upregulates the expression of OPN. (A) The promoter activity of OPN was examined by luciferase reporter gene assay in HepG2-X (or H7402-X) cells, which was abolished by RNAi targeting HBx mRNA, using the indicated doses of pSilencer3.0-X (pSi-HBx) plasmid (**P<0.01 Student's t test). (B) The protein expression of OPN was detected in HepG2-X (or H7402-X) cells by western blot analysis, which was attenuated by pSi-HBx plasmid in a dose-dependent manner. (TIF) [file pone.0031458.s001.tif]

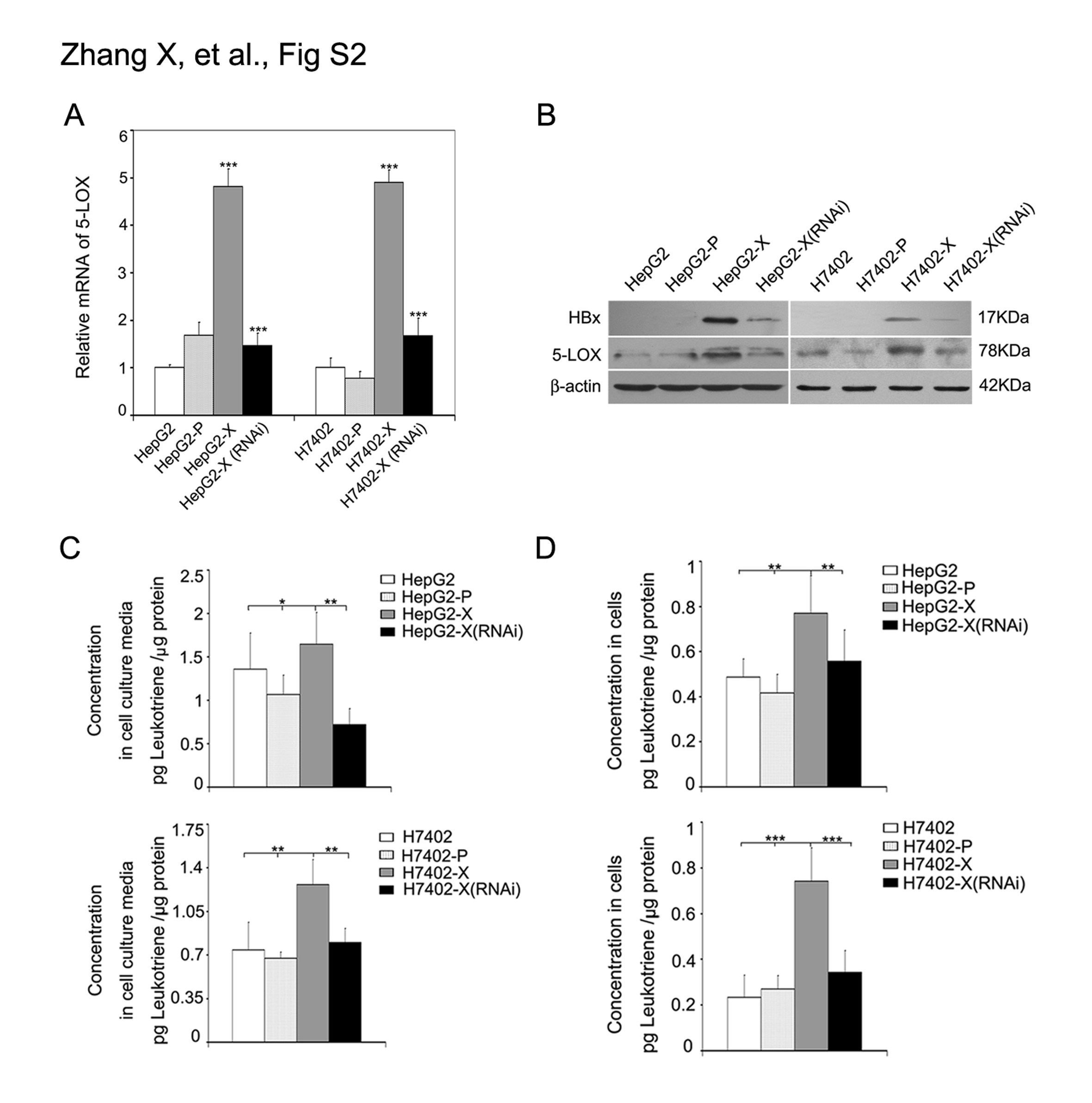

Supplement: Figure S2 — HBx upregulates the expression of 5-LOX. (A) The expression of 5-LOX was examined by real-time PCR in HepG2-X (or H7402-X) cells, which was abolished by using pSilencer3.0-X plasmid (***P<0.001, Student's t test). (B) The expression level of 5-LOX and HBx were detected by western blot analysis. (C,D) The level of LTB4, a metabolite of 5-LOX, was determined by ELISA in conditioned media or in cell lysates from HepG2-X (or H7402-X) cells (*P<0.05, **P<0.01, ***P<0.00l, Student's t test). (TIF) [file pone.0031458.s002.tif]

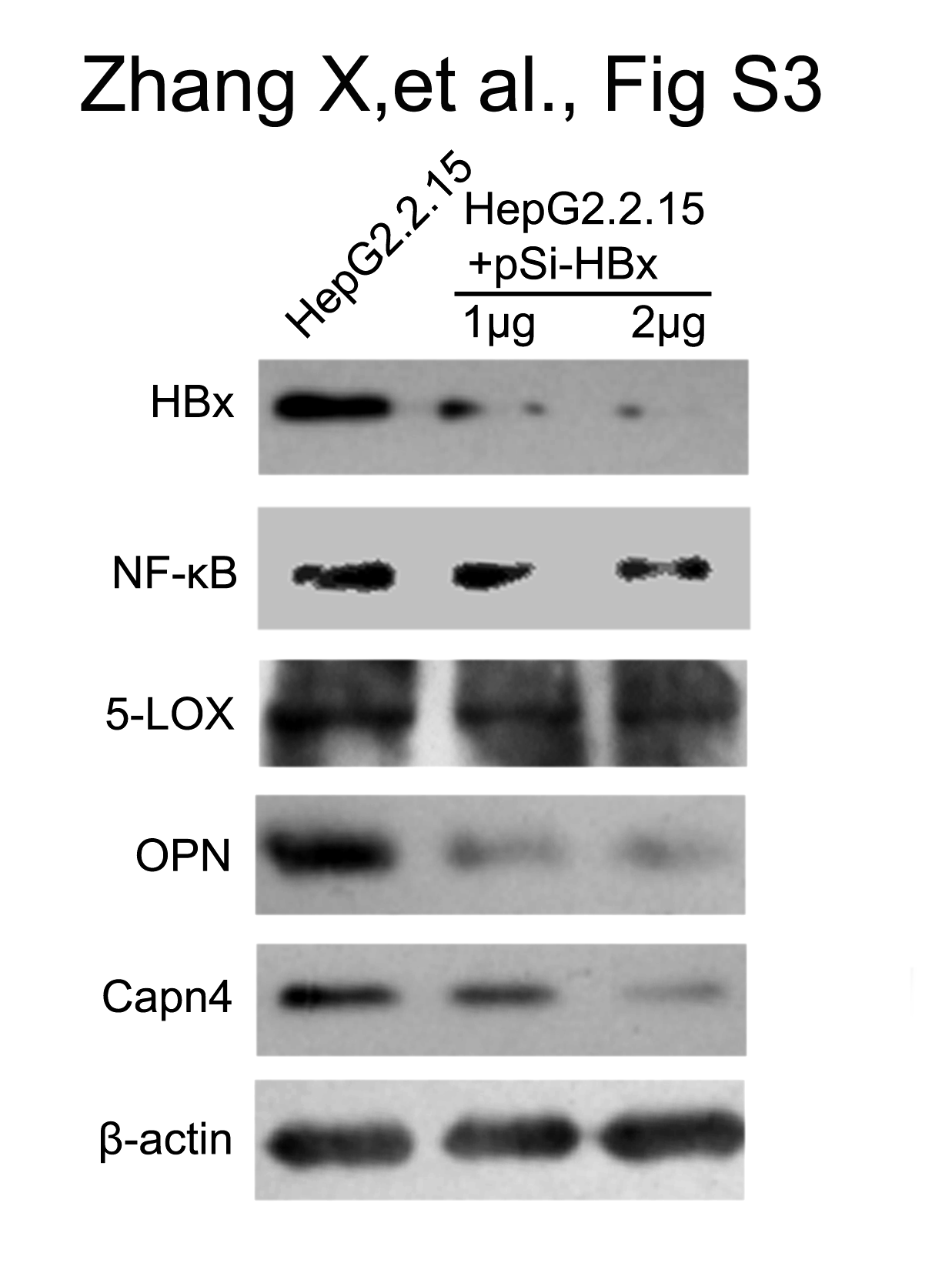

Supplement: Figure S3 — HBx upregulates NF-κB, 5-LOX, OPN and Capn4 in HepG2.2.15 cells. The expression levels of NF-κB, 5-LOX, OPN, Capn4 and HBx were detected by western blot analysis. (TIF) [file pone.0031458.s003.tif]

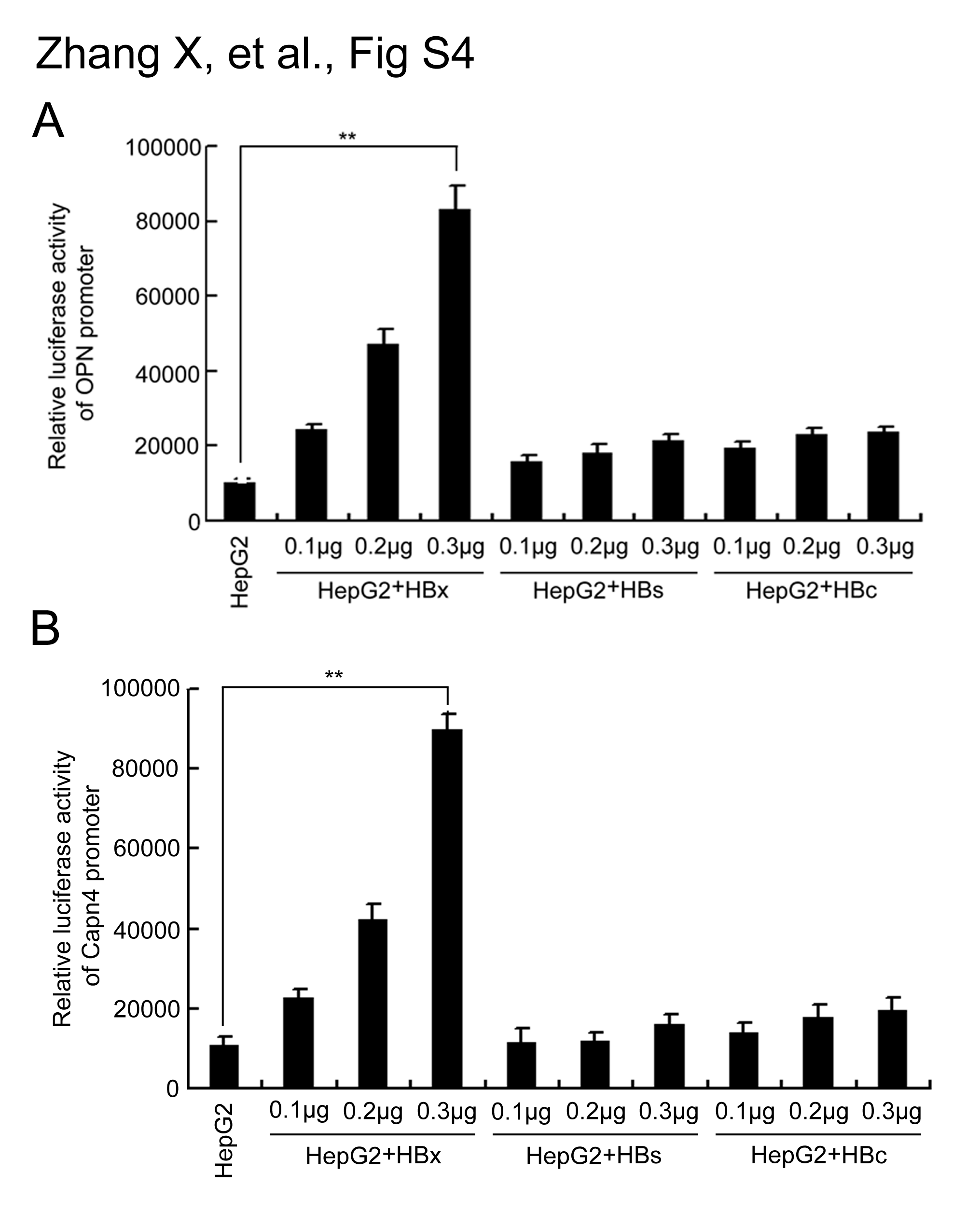

Supplement: Figure S4 — HBx increases the promoter activity of OPN and Capn4, but not by HBc and HBs. (A) The promoter activity of OPN was examined by luciferase reporter gene assay in HepG2 cells (**P<0.01 Student's t test). (B) The promoter activity of Capn4 was examined by luciferase reporter gene assay in HepG2 cells (**P<0.01 Student's t test). (TIF) [file pone.0031458.s004.tif]

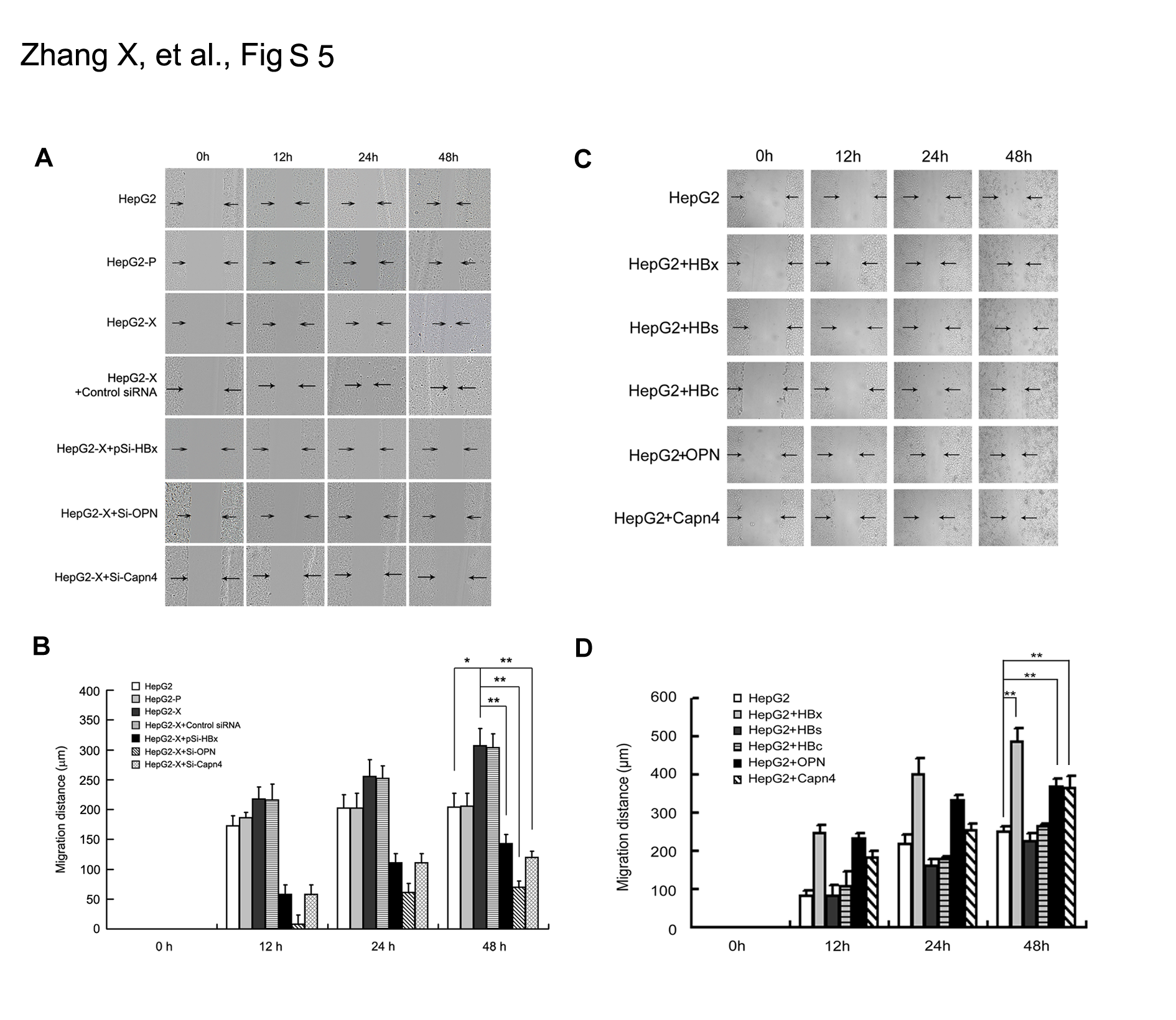

Supplement: Figure S5 — HBx promotes hepatoma cell migration through OPN and Capn4, but not by HBc and HBs. (A) The migration ability of hepatoma cells was examined by wound healing assay when the cells were treated by pSi-HBx, Si-OPN or Si-Capn4. Black arrows indicate the wound edge closure of monolayer cells. (B) The average migration distances of the wound edge were measured in three independent experiments (*P<0.05, **P<0.01, Student's t test). (C) The migration ability of hepatoma cells was examined by wound healing assay when HBx, HBs, HBc, OPN and Capn4 were overexpressed in the cells. Black arrows indicate the wound edge closure of monolayer cells. (D) The average migration distances of the wound edge were measured in three independent experiments (*P<0.05, **P<0.01, Student's t test). (TIF) [file pone.0031458.s005.tif]

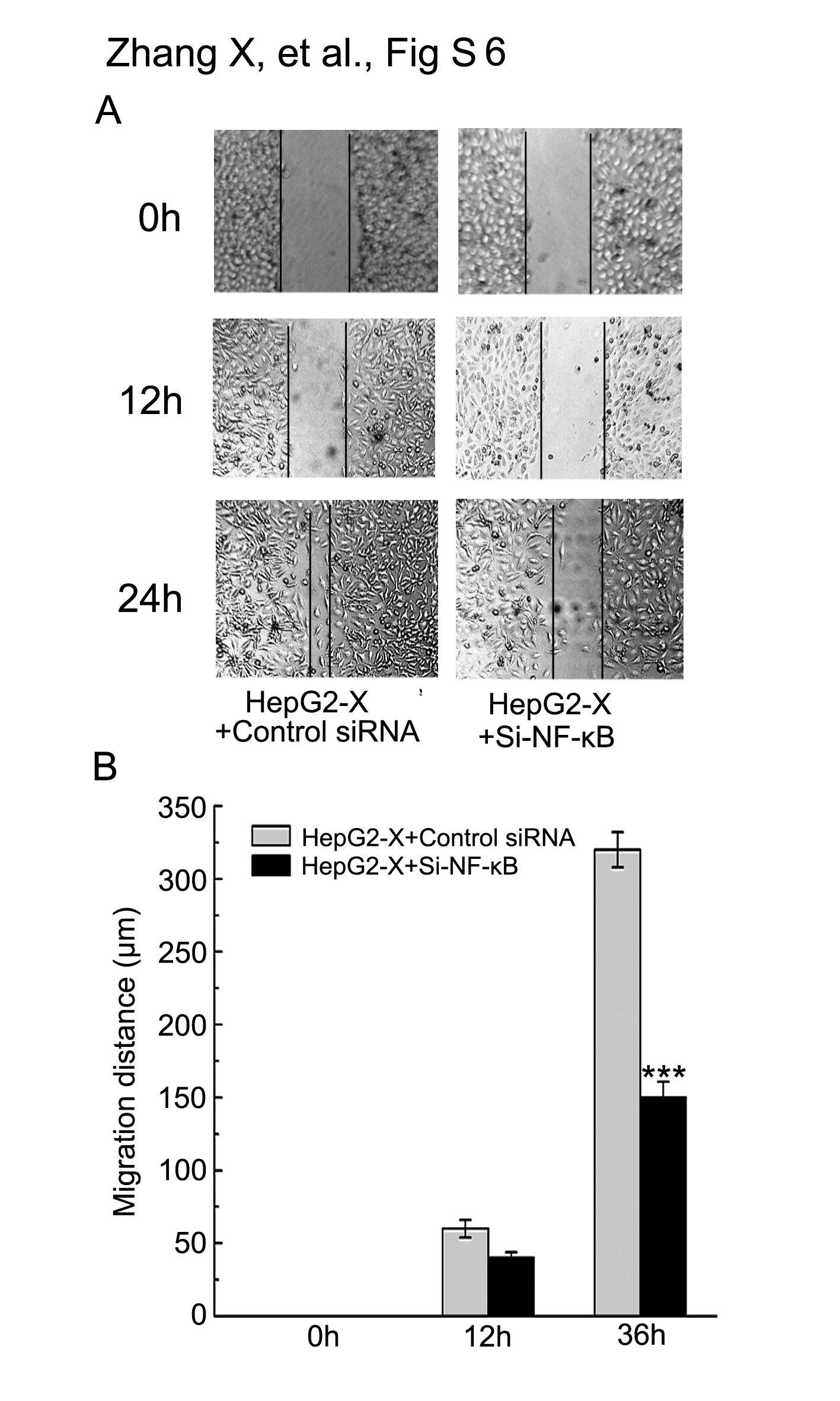

Supplement: Figure S6 — HBx promotes cell migration through NF-κB. (A) The migration ability of hepatoma cells was examined by wound healing assay when the cells were treated with Si-NF-κB. (B) The average migration distances of the wound edge were measured in three independent experiments (***P<0.001, Student's t test). (TIF) [file pone.0031458.s006.tif]
