# Supplementary material for: Hepatitis B Virus X Protein Drives Multiple Cross-Talk Cascade Loops Involving NF-κB, 5-LOX, OPN and Capn4 to Promote Cell Migration
Source: PLoS One. 2012 Feb 15;7(2):e31458. doi: 10.1371/journal.pone.0031458 (PMC3280298; doi:10.1371/journal.pone.0031458)
Supplement: Table S1 — List of primers used for PCR analysis. (DOC) [file pone.0031458.s007.doc]

**Table S1 List of primers used for PCR analysis**

| Genes | NM | Forward (5' to 3') | Reverse (5' to 3') | Product (bp) |
| --- | --- | --- | --- | --- |
| HBx | AB104894 | ATGGCTGCTAGGGTGTGCTG | CTAGGCAGAGGTGAAAAAGTTGC | 465 |
| Capn4 | NM_001749 | CCCCCACGCACACATTACTCCA | CGCTATCCATCACGGCCACCAT | 213 |
| OPN promoter | S78410 | ATGGTACCTAGCGGGTCATTGTTGGGAA | ATCTCGAGTTGGCTGAGAAGGCTGCAA | 2182 |
| OPN  fragment | D28759 | CCCTTCCAAGTAAGTCCAA | TGATGTCCTCGTCTGTAGC | 351 |
| Full-length OPN | D28759 | GCGAATTCATGAGAATTGCAGTGATTTG | ATCTCGAGATTGACCTCAGAAGATGCA | 942 |
| Full-length Capn4 | NM_001003962.1 | CGGGGTACCATGTTCCTGGTTAACTCGTTC | CCGCTCGAGTCAGGAATAC ATAGTCAGCT GC | 801 |
| 5-LOX | NM_000698 | CCCGGGGCATGGAGAGCA | GCGGTCGGGCAGCGTGTC | 415 |
| NF-κB/p65 | NM_021975.2 | GTTCACAGACCTGGCATCC | TGTCACTAGGCGAGTTATAGC | 130 |
| GAPDH | NM_002046 | GGTCATCCCTGAGCTGAACG | TCCGTTGTCATACCAGGAAAT | 298 |
