# Supplementary material for: Hepatitis B Virus X Protein Drives Multiple Cross-Talk Cascade Loops Involving NF-κB, 5-LOX, OPN and Capn4 to Promote Cell Migration
Source: PLoS One. 2012 Feb 15;7(2):e31458. doi: 10.1371/journal.pone.0031458 (PMC3280298; doi:10.1371/journal.pone.0031458)
Supplement: Text S1 — Supporting information. (DOC) [file pone.0031458.s008.doc]

**Supporting information**

**Materials and Methods**

**Cell culture**

HepG2.2.15 (stably transfected with full-length HBV DNA) were maintained in Dulbecco’s modified Eagle’s (DMEM) medium (Gibco, USA) supplemented with heat inactivated 10% fetal calf serum (FCS), 100 U/ml penicillin, and 100 mg/ml streptomycin in a humidified atmosphere of 5% CO_2_ and 95% air at 37℃.

**Construction of the human HBc and HBs eukaryotic expression plasmid**

Total RNA was isolated from HepG2.2.15 cells using Trizol reagent (Invitrogen, USA). Reverse transcription was carried out according to the manufacturer’s instructions. The HBc gene was cloned into the *EcoRI* and *XhoI* sites of the eukaryotic expression vector pcDNA3.0, termed pcDNA-HBc. The HBs gene was cloned into the *EcoRI* and *XhoI* sites of the eukaryotic expression vector pcDNA3.0, termed pcDNA-HBs.

**Wound healing assay**

Cells were seeded in 6-well plates and grown to about 90% confluence before wounding with a 200 µl plastic tip across the monolayer of cells. Debris was removed by washing three times with PBS, and cells were cultured in fresh DMEM medium. Images were captured immediately at 0, 12, 24, and 48 h post-wounding. The migration distance was calculated with the following formula: migration distance = (wound width at the beginning–wound width at each time point)/2 (μm). Each experiment was performed in triplicate. Cells transfected with the control siRNA were used as a negative control.
